# A new genus and species of Tettigarctidae from the Mesozoic of northeastern China (Insecta, Hemiptera, Cicadoidea)

**DOI:** 10.3897/zookeys.632.10076

**Published:** 2016-11-16

**Authors:** Yan Zheng, Jun Chen, Xiaoli Wang

**Affiliations:** 1 Institute of Geology and Paleontology, Linyi University, Shuangling Rd., Linyi 276000, China; 2 State Key Laboratory of Palaeobiology and Stratigraphy, Nanjing Institute of Geology and Palaeontology, East Beijing Road, Nanjing 210008, China

**Keywords:** Coloration pattern, Daohugou, Tettigarctidae, taxonomy

## Abstract

A new genus *Maculaprosbole* of Tettigarctidae with a new species *Maculaprosbole zhengi* is described based on a complete fossil forewing from the Mesozoic of northeastern China. Due to its broad costal area and clavus, *Maculaprosbole zhengi* **gen.** et **sp. n.** can be attributed to the subfamily Cicadoprosbolinae. This genus is similar to the genera *Sanmai* and *Hirtaprosbole* in coloration pattern and forewing venation, respectively. However, it differs from *Hirtaprosbole* in crossvein r-m absent and apical CuA section strongly curved, running along the nodal line for a distance, and *Sanmai* in transverse coloration mainly focusing on the postnodal area. Herein, the prominent coloration pattern of this new taxon is discussed.

## Introduction

Tettigarctidae, the most primitive family of Cicadoidea, is now relict with only two modern species attributed into one genus (Moulds 1990, 2012, Shcherbakov 2009). The taxonomy of the Tettigarctidae is based mainly on the fore- and hindwing venation (Shcherbakov 2009, Li et al. 2012), and this family is divided into two subfamilies based on forewing features: Cicadoprosbolinae Evans, 1956 and Tettigarctinae Distant, 1905 (Wang B and Zhang 2009, Li et al. 2012). The fossils records of the Mesozoic Tettigarctidae are rather diverse (Nel 1996, Nel et al. 1998, Dietrich 2002, Menon 2005, Moulds 2005, Wang B and Zhang 2009, Wang B et al. 2013). The earliest Tettigarctidae appeared in the earliest Jurassic and terminal Triassic (ca. 200 Myr) of Eurasia (Shcherbakov 2009). The *Paratettigarcta zealandica* at around 23 ~ 16 Ma is known as the youngest Tettigarctidae fossil (Kaulfuss and Moulds 2015). Up to now, 27 genera and 40 extinct species (Liu et al. 2015, Kaulfuss and Moulds 2015, Chen and Wang B 2016, Chen et al. 2016) of Tettigarctidae have been reported from all over the world, ranging from the Late Triassic to the Eocene, and are distributed from Northern Hemisphere to Southern Hemisphere, Eurasia, Australia, Africa, and England, etc (Shcherbakov and Popov 2002, Martins-Neto et al. 2003, Wappler 2003, Shcherbakov 2009). Beyond that, two living species within one genus of Tettigarctidae are restricted to high altitude habitat in continental South Australia and Tasmania (Carver et al. 1992, Moulds 1990, Moulds 2005, Li et al. 2012, Liu et al. 2016).

A large number of fossils, especially the highly diverse array of insects, have been well-known and described from Daohugou based on the exceptionally well-preserved materials, showing sharp details of morphology, taxonomy and evolution (Rasnitsyn et al. 2006, Pott et al. 2012, Wang B et al. 2013, Chen et al. 2014, Wang H et al. 2015). To date, 25 insect orders have been reported from the Daohugou Biota (Huang 2010, Li et al. 2010, 2013, Wang B et al. 2013). In Tettigarctidae, eleven species within seven genera had been described from Daohugou to date (Wang B and Zhang 2009, Li et al. 2012, Chen et al. 2014, Chen et al. 2016, Chen and Wang B 2016, this study). The Tettigarctidae, in fact, is a particular group which is known to be much more rich in Daohugou than in any other fauna (Wang B and Zhang 2009, Wang B et al. 2013, Chen et al. 2016). However, their systematic position is still not very clear (Wang B and Zhang 2009, Li et al. 2012, Liu et al. 2016).

In this paper, a new fossil genus is confirmed and described, with a new species of the Tettigarctidae from Daohugou in northeast China.

## Material and methods

The fossil specimen studied herein was collected from the Middle Jurassic Daohugou deposits (41°18.31'N; 119°13.18'E) in Ningcheng Country, Chifeng City, Inner Mongolia of China. Very recently some studies indicate Daohugou is enjoyed a humid and warm-tempterate climate in the Middle Jurassic based on the palaeoenvironmental reconstructions (Ren and Krzeminski 2002, Wang B et al. 2013, Na et al. 2015). This type fossil is preserved as impressions on the surface of grey tuffaceous siltstones. The material described in this paper is deposited in Shandong Tianyu Museum of Nature at Pingyi, Shandong province, China.

The fossil was examined and then photographed with the Nikon D800 digital camera and the Photomicrographs were taken with a Nikon SMZ1000 stereomicroscope. The line drawing was created using Adobe Illustrator CS3 and Adobe Photoshop CS5. The quantitatively measure of forewing used NIH ImageJ software (http://rsb.info.nih.gov/ij/). The terminologies of wing venation and cell nomenclature used in herein are modified after Chen et al. (2015). Venation symbols: main longitudinal veins are SC, ScP, RA, RP, M, CuA, CuP and A; crossveins are ir, im, m-cu; cells are a1 ~ a11. The norms of measurements for the wing were following: the wing length measured from the base to the apex and the width measured at the widest part (Li et al. 2012).

## Systematic paleontology

### Order Hemiptera L., 1758 Suborder Cicadomorpha Evans, 1946 Superfamily Cicadoidea Latreille, 1802 Family Tettigarctidae Distant, 1905 Subfamily Cicadoprosbolinae Evans, 1956

#### Maculaprosbole gen. n.

Taxon classificationAnimaliaHemipteraTettigarctidae

http://zoobank.org/947E0D09-5577-417A-A191-5E12A3A9CBE0

##### Type species.

**Figure 1.**
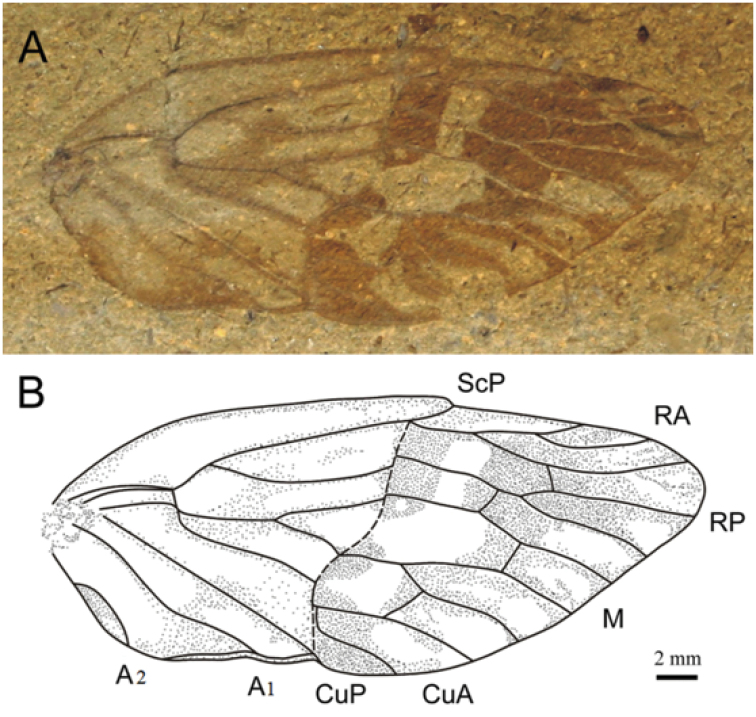
Holotype of *Maculaprosbole zhengi* gen. et sp. n. **A** Photograph **B** Line drawing.

*Maculaprosbole zhengi* new species, designated herein (Fig. 1). No other species are currently included in the genus.

##### Etymology.

The generic name is a composition of the Latin “macula”, meaning spots and stripes, and the suffix of the genus of *Cicadoprosbole* Becker-Migdisova, 1947.

##### Diagnosis.

Forewing large-sized, relatively wide and with oblique apical margin. Wing membrane with distinct color patterns. Clavus and costal area long. Nodal line at the middle of wing. Nodus distinct. RA with three branches; crossvein ir halfway from nodal line to wing tip; vein RP single and extended upward along the nodal line; vein M four-branched; M_1+2_ branched beyond M_3+4_; M_1+2_ fork into M_1_ and M_2_ with a right angle, and M_1_ fused with RP for a distance, then nearly parallel to M_2_; crossvein r-m absent; crossvein m nearly straight, almost perpendicular to M_2_; vein CuA strongly downward along nodal line, branching into CuA_1_ and CuA_2_ just after nodal line; vein CuP almost straight, ending at about 2/5 of wing.

##### Remarks.

The new genus undoubtedly belongs to the family Tettigarctidae based on the following diagnostic characteristics of the forewing: nodal line clearly visible; vein RA three-branched; intercostal area widest beyond nodal line; vein RP single and strongly bowed; vein M four-branched. The stem of M is shorter than ScP+R. *Maculaprosbole* gen. n. is assigned to the subfamily Cicadoprosbolinae based on the forewing features: costal area broad, basal cell narrow; clavus arched; branch CuA_2_ long, sinuous and near S-shaped. This genus is most similar in coloration pattern of forewing (such as dark or light speckles, longitudinal stripes) with *Sanmai* Chen, Zhang & Wang B 2016, but differs from *Sanmai* in the transverse coloration mainly focusing on the postnodal area. *Maculaprosbole* shares some features in forewing venation with that of *Hirtaprosbole* Liu, Li & Yao, 2015: nodal line at middle of forewing, RA three-branched; stem ScP+R longer than stem M; CuP straight; cell a6 nearly quadrate; cell a8 subequal to cell a10 in length. However, it differs from *Hirtaprosbole* in the following characters: stem ScP+RA separated at the nodal line, ScP ending beyond the middle of anterior margin M_1_ fused with RP for a distance and crossvein r-m absent (vs. r-m is located between M_1_ and RP); apical CuA section strongly curved, running along nodal line for a distance (vs. CuA slightly sigmoidal, not along nodal line).

#### Maculaprosbole zhengi sp. n.

Taxon classificationAnimaliaHemipteraTettigarctidae

http://zoobank.org/F16A76A5-C78B-40C4-AC9A-3169B02046CF

Fig. 1

##### Diagnosis.

As for genus.

##### Description.

Forewing long and elongate apically and relatively wide, with oblique apical margin, near triangular in the tip, with distinctly dark or gray pigmented transverse bands, irregular speckles and longitudinal stripes, mainly behind the nodal line and postnodal area. Length about 34.04 mm, width about 14.54 mm, with the ratio of length/width approximately 2.34; costal margin broad, length about 21.36 mm; clavus arched, small and broad (length 15.06 mm, maximum width 4.55 mm), with conspicuous light pigmented bands. Nodal line situated in the middle of forewing. Crossvein r-m absent; branched into ScP+R and M at basal 0.17 wing length. Stem ScP+R bifurcated into ScP+RA and RP at basal 0.47 wing length; vein ScP forked with RA at nodal line, and terminating at nodus; RA with three branches, RA_1_ short and nearly straight, RA_2_ and RA_3_ long and slightly sinuous, RA_2_ parallel to RA_3_; branch RA_3_ connected with vein RP by the crossvein ir. Crossvein ir at the middle of nodal line and outer margin. Vein RP strongly curved, running along the nodal line for a distance; vein M_1_ strongly curved and fused with RP for a distance, then subparallel to M_2_; stem ScP+R relatively shorter than stem M. Stem M forked into M_1+2_ and M_3+4_ at basal 0.15 wing length, and at different level. M_1+2_ branched into M_1_ and M_2_ at basal 0.33 wing length; M_3+4_ bifurcated into M_3_ and M_4_ at basal 0.21 wing length; stem CuA long and initially sinuous, fusing with nodal line and running along with nodal line for a distance, then branched into CuA_1_ and CuA_2_ just beyond nodal line. CuA_1_ long and relatively straight; CuA_2_ short and obviously sinuous; CuP long and straight. A_1_ sinuous. A_2_ short and strongly curved; eleven apical cells.

##### Etymology.

The species name refers to Prof. Xiaoting Zheng, who is the founder of Shandong Tianyu Museum of Nature and donated the type material.

##### Type specimen.

Holotype STMN48-1813, complete forewing; housed in Shandong Tianyu Museum of Nature.

##### Locality and age.

Middle Jurassic; Daohugou Village, Ningcheng County, Chifeng City, Inner Mongolia, China.

## Discussion

The Daohugou palaeolake was a low-energy preservational environment (Wang B et al. 2009, Chen et al. 2014, 2016). A large number of insect fossils have been found with well-preserved body structure and wing impression in the fossil beds (Ren et al. 2002, Wang B et al. 2013). Wang B et al. (2013) reported approximately 9% Mesozoic cicadomorph fossils designated as Jurassic tettigarctids. Tettigarctidae is quite abundant and morphologically diversified in the Daohugou area (Wang B and Zhang 2009, Chen et al. 2016, Chen and Wang B 2016). To date, one species within the genus *Sunotettigarcta* of the subfamily Tettigarctinae and nine species within five genera (*Macrotettigarcta*, *Shuraboprosbole*, *Tianyuprosbole*, *Hirtaprosbole* and *Sanmai*) assigned to the subfamily Cicadoprosbolinae have been described and illustrated (Wang B and Zhang 2009, Li et al. 2012, Chen et al. 2014, 2016, Chen and Wang B 2016). Those fossils provide new insights into the evolution, ecology, and behavior of tettigarctids. We herein attribute a new genus *Maculaprosbole* to the family Tettigarctidae. Material of *Maculaprosbole* is undoubtedly identified as a new taxon and distinctly differs from other tettigarctids in possessing these forewing characters: nodal line at the middle of wing; vein RA divided into three branches; vein RP running along the nodal line for a distance and fused with M_1_ for a long distance; CuA strongly curved, fused with nodal line for a distance and forked into CuA_1_ and CuA_2_ just beyond it. This study brings new insights to improve our knowledge of the biodiversity and wing structure diversification of the Mesozoic Tettigarctidae.

Nowadays, the prominent color pattern on wings, with dark or light stripes and conspicuous transverse longitudinal bands, is a topic that has been known in many insect fossils (Cott 1940, Wang Y et al. 2010, Chen et al. 2016). The color patterns on wings provide camouflage by strongly contrasting markings such as spots or stripes to hide themselves or frighten predators (Stevens et al. 2006; Stevens and Merilaita 2009; Seymoure and Aiello 2015), and are also attributed to sexual selection on visual signals (Wang B et al. 2006, Punzalan et al. 2008, Hilfert-Rüppell and Rüppell 2013).

In Mesozoic tettigarctids, eight species with four genera (*Sanmai*, *Protabanus*, *Liassocicada* and *Shuraboprosbole*) have been reported possessing a color pattern with dark or light stripes and irregularly colored bands (Hong 1982, Nel 1996, Wang B and Zhang 2009, Kaulfuss and Moulds 2015, Chen et al. 2016). The disruptive coloration of the forewing seems be an autapomorphy of *Maculaprosbole*. The new fossil has prominent disruptive coloration of the type with dark or light speckles and longitudinal stripes on the forewing membrane, which is remarkably different from most Mesozoic tettigarctids. However, this disruptive coloration pattern seems to be similar to *Sanmai* in the Daohugou beds. The stripes and spots on the forewings of *Sanmai* and *Maculaprosbole* might be effective color camouflage and break up the body outline as well as the surface (Cuthill et al. 2005, Schaefer and Stobbe 2006, Chen et al. 2016).

## Supplementary Material

XML Treatment for Maculaprosbole

XML Treatment for Maculaprosbole zhengi

## References

[B1] Carver M Gross GF Woodward TE (1992) Hemiptera. In: Naumann ID (Ed.) Insects of Australia. CSIRO and Melbourne University Press, 429–509.

[B2] Chen J Wang B (2016) A giant tettigarctid cicada from the Mesozoic of northeastern China. Spixiana 39(1): 119–124.

[B3] Chen J Wang B Zhang H Wang X (2014) A remarkable new genus of Tettigarctidae (Insecta, Hemiptera, Cicadoidea) from the Middle Jurassic of northeastern China. Zootaxa 3764(5): 581–586. doi: 10.11646/zootaxa.3764.5.6 24870657 10.11646/zootaxa.3764.5.6

[B4] Chen J Wang B Zhang H Wang X Zheng X (2015) New fossil Procercopidae (Hemiptera: Cicadomorpha) from the Middle Jurassic of Daohugou, Inner Mongolia, China. European Journal of Entomology 112(2): 373. doi: 10.14411/eje.2015.044

[B5] Chen J Zhang H Wang B Zheng Y Wang X Zheng X (2016) New Jurassic tettigarctid cicadas from China with a novel example of disruptive coloration. Acta Palaeontologica Polonica. doi: 10.4202/app.00238.2015

[B6] Cott HB (1940) Adaptive coloration in animals. Methuen, London, 508 pp http://afrilib.odinafrica.org/handle/0/1815

[B7] Cuthill IC Stevens M Sheppard J Maddocks T Párraga CA Troscianko TS (2005) Disruptive coloration and background pattern matching. Nature 434(7029): 72–74. doi: 10.1038/nature03312 15744301 10.1038/nature03312

[B8] Dietrich CH (2002) Evolution of Cicadomorpha (Insecta, Hemiptera). Denisia 176: 155–170.

[B9] Hong Y (1982) Mesozoic Fossil Insects of Jiuquan Basin in Gansu Province. Geological Publishing House, Beijing, 223 pp [In Chinese]

[B10] Huang D (2010) Diversity of Jurassic insects–exemplified by Daohugou fauna. Earth Science Frontiers 17: 149–150.

[B11] Hilfert-Rüppell D Rüppell G (2013) Do coloured-winged damselflies and dragonflies have flight kinematics different from those with clear wings?. International Journal of Odonatology 16(2): 119–134. doi: 10.1080/13887890.2013.763332

[B12] Kaulfuss U Moulds M (2015) A new genus and species of tettigarctid cicada from the early Miocene of New Zealand: Paratettigarcta zealandica (Hemiptera, Auchenorrhyncha, Tettigarctidae). ZooKeys 484: 83–94. doi: 10.3897/zookeys.484.8883 10.3897/zookeys.484.8883 PMC4361785 25829843

[B13] Li S Wang Y Ren D Pang H (2012) Revision of the genus *Sunotettigarcta* Hong, 1983 (Hemiptera, Tettigarctidae), with a new species from Daohugou, Inner Mongolia, China. Alcheringa: An Australasian Journal of Palaeontology 36: 501–507. doi: 10.1080/03115518.2012.680722

[B14] Li S Wang Y Ren D Szwedo J Pang H (2010) Froghoppers, leafhoppers, planthoppers and their allies from the Mesozoic of Northeastern China (Hemiptera: Cicadomorpha and Fulgoromorpha). Earth Science Frontiers 17: 250–251.

[B15] Liu XH Li Y Yao YZ Ren D (2016) A hairy-bodied tettigarctid (Hemiptera: Cicadoidea) from the latest Middle Jurassic of northeast China. Alcheringa: An Australasian Journal of Palaeontology 40: 1–7. doi: 10.1080/03115518.2016.1145390

[B16] Martins-Neto RG Gallego OF Melchor RN (2003) The Triassic insect fauna from South America (Argentina, Brazil and Chile): a checklist (except Blattoptera and Coleoptera) and descriptions of new taxa. Acta Zoologica Cracoviensia 46: 229–256.

[B17] Menon F (2005) New record of Tettigarctidae (Insecta, Hemiptera, Cicadoidea) from the Lower Cretaceous of Brazil. Zootaxa 1087: 53–58. http://www.mapress.com/zootaxa/

[B18] Moulds MS (1990) Australian Cicadas. New South Wales University Press, Kensington, 217.

[B19] Moulds MS (2005) An appraisal of the higher classification of cicadas (Hemiptera: Cicadoidea) with special reference to the Australian fauna. Records of the Australian Museum 57: 375–446. doi: 10.3853/j.0067-1975.57.2005.1447

[B20] Moulds MS (2012) A review of the genera of Australian cicadas (Hemiptera: Cicadoidea). Zootaxa 3287: 1–262.

[B21] Na Y Manchester SR Sun C Zhang S (2015) The Middle Jurassic palynology of the Daohugou area, Inner Mongolia, China, and its implications for palaeobiology and palaeogeography. Palynology 39(2): 270–287. doi: 10.1080/01916122.2014.961664

[B22] Nel A (1996) Un Tettigarctidae fossile du Lias Européen (Cicadomorpha, Cicadoidea, Tettigarctidae). Ecole pratique des hautes Etudes, Biologie et Evolution des Insectes 9: 83–94.

[B23] Nel A Zarbout M Barale G Philippe M (1998) *Liassotettigarcta africana* sp. n. (Auchenorrhyncha: Cicadoidea: Tettigarctidae), the first Mesozoic insect from Tunisia. European Journal of Entomology 95: 593–598.

[B24] Pott C McLoughlin S Wu S Friis EM (2012) Trichomes on the leaves of *Anomozamites villosus* sp. n. (Bennettitales) from the Daohugou beds (Middle Jurassic), Inner Mongolia, China: Mechanical defence against herbivorous arthropods. Review of Palaeobotany and Palynology 169: 48–60. doi: 10.1016/j.revpalbo.2011.10.005

[B25] Punzalan D Rodd FH Rowe L (2008) Sexual selection mediated by the thermoregulatory effects of male colour pattern in the ambush bug *Phymata americana*. Proceedings of the Royal Society of London B: Biological Sciences 275(1634): 483–492. doi: 10.1098/rspb.2007.1585 10.1098/rspb.2007.1585 PMC2596820 18089533

[B26] Rasnitsyn AP Zhang H Wang B (2006) Bizarre fossil insects, the web-spinning sawflies of the genus *Ferganolyda* (Vespida, Pamphilioidea) from the Middle Jurassic of Daohugou, Inner Mongolia, China. Palaeontology 49: 907–916. doi: 10.1111/j.1475-4983.2006.00574.x

[B27] Ren D krzeMinski W (2002) Eoptychopteridae (Diptera) from the Middle Jurassic of China. In Annales zoologici 52: 207–210.

[B28] Ren D Gao KQ Guo ZG Ji SA Tan JJ Song Z (2002) Stratigraphic division of the Jurassic in the Daohugou Area, Ningcheng, Inner Mongolia. Geological Bulletin of China 21: 584–591.

[B29] Schaefer HM Stobbe N (2006) Disruptive coloration provides camouflage independent of background matching. Proceedings of the Royal Society of London B: Biological Sciences 273(1600): 2427–2432. doi: 10.1098/rspb.2006.3615 10.1098/rspb.2006.3615 PMC1634905 16959631

[B30] Seymoure BM Aiello A (2015) Keeping the band together: evidence for false boundary disruptive coloration in a butterfly. Journal of evolutionary biology 28(9): 1618–1624. doi: 10.1111/jeb.12681 26109438 10.1111/jeb.12681

[B31] Shcherbakov DE (2009) Review of the fossil and extant genera of the cicada family Tettigarctidae (Hemiptera: Cicadoidea). Russian Entomological Journal 17: 343–348.

[B32] Shcherbakov DE Popov YA (2002) Superorder Cimicidea Laicharting, 1781 order Hemiptera Linné, 1758. The bugs, cicadas, plantlice, scale insects, etc. In: Rasnitsyn AP Quicke DLJ (Eds) History of Insects. Kluwer Academic Publisher, Dordrecht, 152–155.

[B33] Stevens M Merilaita S (2009) Defining disruptive coloration and distinguishing its functions. Philosophical Transactions of the Royal Society of London B: Biological Sciences 364(1516): 481–488. doi: 10.1098/rstb.2008.0216 18990673 10.1098/rstb.2008.0216 PMC2674077

[B34] Stevens M Cuthill IC Windsor AM Walker HJ (2006) Disruptive contrast in animal camouflage. Proceedings of the Royal Society of London B: Biological Sciences 273(1600): 2433–2438. doi: 10.1098/rspb.2006.3614 10.1098/rspb.2006.3614 PMC1634902 16959632

[B35] Wang B Zhang H (2009) Tettigarctidae (Insecta: Hemiptera: Cicadoidea) from the Middle Jurassic of Inner Mongolia, China. Geobios 42(2): 243–253. doi: 10.2110/palo.2012.p12-045r

[B36] Wang B Zhang HC Fang Y Zhang YT (2008) A revision of Palaeontinidae (Insecta: Hemiptera: Cicadomorpha) from the Jurassic of China with descriptions of new taxa and new combinations. Geological Journal 43(1): 1–18. doi: 10.1002/gj.1092

[B37] Wang B Li J Fang Y Zhang H (2009) Preliminary elemental analysis of fossil insects from the Middle Jurassic of Daohugou, Inner Mongolia and its taphonomic implications. Chinese Science Bulletin 54(5): 783–787. doi: 10.1007/s11434-008-0561-5

[B38] Wang B Szwedo J Zhang H (2012) New Jurassic Cercopoidea from China and their evolutionary significance (Insecta: Hemiptera). Palaeontology 55(6): 1223–1243. doi: 10.1111/j.1475-4983.2012.01185.x

[B39] Wang B Zhang H Fang Y Zhang Z (2006) A new genus and species of Palaeontinidae (Insecta: Hemiptera) from the Middle Jurassic of Daohugou, China. Annales Zoologici 56(4): 757–762. doi: 10.3161/000345406779508606

[B40] Wang B Zhang H Jarzembowski EA Fang Y Zheng D (2013) Taphonomic variability of fossil insects: a biostratinomic study of Palaeontinidae and Tettigarctidae (Insecta: Hemiptera) from the Jurassic Daohugou Lagerstätte. Palaios 28(4): 233–242. doi: 10.2110/palo.2012.p12-045r

[B41] Wang H Li S Zhang Q Fang Y Wang B Zhang H (2015) A new species of Aboilus (Insecta, Orthoptera) from the Jurassic Daohugou beds of China, and discussion of forewing coloration in Aboilus. Alcheringa: An Australasian Journal of Palaeontology 39(2): 250–258. doi: 10.1080/03115518.2015.993297

[B42] Wang Y Shih C-K Li S Ren D (2010) Homoptera: 17 years underground. In: Ren D Shih C-K Gao T Yao Y Zhao Y (Eds) Silent Stories – Insect Fossil Treasures from Dinosaur Era of the Northeastern China. Science Press, Beijing, 118–138.

[B43] Wappler T (2003) Systematik, Phylogenie, Taphonomie und Palaookologie der Insekten aus dem Mittle Eowan des Eckfelder marares. Clausthaler Geozissenchafen, Vulkaneifel, 241 pp.

